# Central Role of Adenosine 5′-Phosphosulfate Reductase in the Control of Plant Hydrogen Sulfide Metabolism

**DOI:** 10.3389/fpls.2018.01404

**Published:** 2018-09-24

**Authors:** Yang Fu, Jun Tang, Gai-Fang Yao, Zhong-Qin Huang, Yan-Hong Li, Zhuo Han, Xiao-Yan Chen, Lan-Ying Hu, Kang-Di Hu, Hua Zhang

**Affiliations:** ^1^School of Food and Biological Engineering, Hefei University of Technology, Hefei, China; ^2^Xuzhou Institute of Agricultural Sciences of the Xuhuai District of Jiangsu Province, Xuzhou, China; ^3^Anhui Province Key Laboratory of Functional Compound Seasoning, Anhui Qiangwang Seasoning Food Co., Ltd., Jieshou, China

**Keywords:** hydrogen sulfide (H_2_S), adenosine 5′-phosphosulfate reductase (APR), sulfur assimilation, transcription factors, gene expression

## Abstract

Hydrogen sulfide (H_2_S) has been postulated to be the third gasotransmitter in both animals and plants after nitric oxide (NO) and carbon monoxide (CO). In this review, the physiological roles of H_2_S in plant growth, development and responses to biotic, and abiotic stresses are summarized. The enzymes which generate H_2_S are subjected to tight regulation to produce H_2_S when needed, contributing to delicate responses of H_2_S to environmental stimuli. H_2_S occupies a central position in plant sulfur metabolism as it is the link of inorganic sulfur to the first organic sulfur-containing compound cysteine which is the starting point for the synthesis of methionine, coenzyme A, vitamins, etc. In sulfur assimilation, adenosine 5′-phosphosulfate reductase (APR) is the rate-limiting enzyme with the greatest control over the pathway and probably the generation of H_2_S which is an essential component in this process. APR is an evolutionarily conserved protein among plants, and two conserved domains PAPS_reductase and Thioredoxin are found in APR. Sulfate reduction including the APR-catalyzing step is carried out in chloroplasts. APR, the key enzyme in sulfur assimilation, is mainly regulated at transcription level by transcription factors in response to sulfur availability and environmental stimuli. The *cis*-acting elements in the promoter region of all the three *APR* genes in *Solanum lycopersicum* suggest that multiple factors such as sulfur starvation, cytokinins, CO_2_, and pathogens may regulate the expression of *SlAPRs*. In conclusion, as a critical enzyme in regulating sulfur assimilation, APR is probably critical for H_2_S generation during plants’ response to diverse environmental factors.

## Introduction

Over centuries, hydrogen sulfide (H_2_S) has only been well-known for its unpleasant smell and fierce toxicity. After the gaseous signals nitric oxide (NO) and carbon monoxide (CO), H_2_S is recently emerging as a multifunctional signaling molecule in animals, and plants. Endogenous H_2_S production has been observed in mammalian cells and shown to control a variety of physiological processes and play important roles in the regulation of the pathogenesis of various diseases (Wang, 2012). In plants, sulfur is an essential macronutrient for growth and development, participating in the synthesis of cysteine, methionine and in many other essential cellular constituents, such as reduced glutathione (GSH) and coenzyme A (Romero et al., 2014). Endogenous H_2_S in plant is generated through the pathway of sulfur assimilation or the decomposition of L-/D-cysteine. L-cysteine desulfhydrase (LCD) catalyzes the production of H_2_S, ammonia and pyruvate with L-cysteine as the substrate, while DCD degrades D-cysteine to generate H_2_S in mitochondria (Rausch and Wachter, 2005). Besides, an O-acetylserine(thiol)lyase homolog DES1 also shows L-Cysteine desulfhydrase activity in Arabidopsis (Álvarez et al., 2010). In sulfur assimilation, sulfite is reduced by sulfite reductase (SiR) to produce H_2_S in chloroplast (Rausch and Wachter, 2005). Adenosine 5′-phosphosulfate reductase (APR) is a rate-limiting enzyme in sulfur assimilation, which controls the flow of inorganic sulfur into cysteine and probably the endogenous production of H_2_S. In recent decades, we have witnessed significant progresses in the functional study of H_2_S and here we reviewed the physiological role of H_2_S in plant, the central role of APR in sulfur assimilation and proposed the future perspectives of APR research.

## Physiological Role of H_2_S in Plant

Accumulating articles show that endogenous H_2_S is involved in various physiological activities in animals including human, such as vasodilation, anti-hypertensive, anti-inflammatory, heart protection, smooth muscle relaxation, promotion of vascular endothelial cell proliferation, and brain development (Wang, 2012). Meanwhile, the past decade has witnessed a growing body of evidence confirming the signaling role of H_2_S in plants as well. Although phytotoxic at high concentrations, low dose of H_2_S has been shown to play important roles in diverse processes of plant life, including plant growth, and development, responses to biotic and abiotic stresses (Zhang et al., 2008; Zhang et al., 2009; Zhang et al., 2010; Chen et al., 2011; Shen et al., 2012, 2013; Jin et al., 2013; Xie et al., 2014). H_2_S improves seed germination and the yield of multiple crops including bean, corn, wheat, and pea (Dooley et al., 2013). H_2_S also interacts with NO, CO and auxin to modulate root formation, both lateral and adventitious roots (Zhang et al., 2009; Lin et al., 2012; Fang H. et al., 2014; Fang T. et al., 2014; Jia et al., 2015). Also, accumulating researches find that H_2_S alleviates diverse abiotic stresses including heavy metal stress, drought and osmotic stresses by improving the antioxidative capacity in plant (Zhang et al., 2008, 2010; Sun et al., 2013; Shi et al., 2014). Salicylic acid (SA) induces endogenous H_2_S production by increasing LCD activity to tolerate Cd stress, while the positive effect of SA is diminished in LCD-knockout *A. thaliana*, suggesting that H_2_S acts downstream of SA in regulating Cd tolerance (Qiao et al., 2015). Stomatal movement is crucial for plant responses to environmental stimuli, and H_2_S is found to interact with ABA in the stomatal regulation responsible for drought stress in *A. thaliana* (Jin et al., 2013). Consistently, Scuffi et al. (2014) observed that ABA failed to close stomata in *des1* mutant which encoding a L-cysteine desulfhydrase, suggesting that endogenous H_2_S is essential for ABA signaling in guard cells. H_2_S also plays a vital role in plant response to biotic stress. In *A. thaliana*, endogenous H_2_S increases when plant is infected with *Pseudomonas syringae* (Shi et al., 2015). Besides, the activity of DES1 is elevated in pathogen-infected plants (Bloem et al., 2004). H_2_S can also delay flower opening and senescence in cut flowers (Zhang et al., 2011). Later the senescence-alleviating effect of H_2_S is found in diverse postharvest fruits and vegetables including strawberry, kiwifruit, grape, apple, banana and broccoli, possibly through improving antioxidative capacity and antagonizing the production of ethylene, a hormone playing a major role in plant senescence, and fruit ripening (Hu et al., 2012; Gao et al., 2013; Li et al., 2014; Ni et al., 2016; Ge et al., 2017). Besides, H_2_S generated in cytosol negatively regulates autophagy and modulates the transcriptional profile of *A. thaliana* by the study of *des1* mutant, whereas the negative regulation of autophagy by sulfide is independent of reactive oxygen species (Álvarez et al., 2012; Laureano-Marín et al., 2016). The multifunctional role of H_2_S in plant growth and development highlights the importance of the regulation of endogenous generation of H_2_S in adaption to growth stages and in response to biotic and abiotic stresses.

## Endogenous Production of H_2_S in Plant and the Central Role of 5′-Adenylylsulfate Reductase in Sulfur Assimilation

Sulfur is essential for all living organisms as a key constituent of the amino acids cysteine and methionine, as well as GSH, several group-transfer coenzymes and vitamins (Romero et al., 2014). Animals require dietary sources of methionine as their cells are not able to assimilate inorganic sulfur. The endogenous generating enzymes of H_2_S in mammals are known as cystathionine-γ-lyase (CSE), cystathionine-β-synthase (CBS), and 3-mercaptopyruvate transsulfatase (3-MST) (Wang, 2012). In contrary to animals, plants reduce and incorporate inorganic sulfur, which is almost entirely available as oxidized sulfate, into cysteine via the reductive sulfate assimilation pathway.

**FIGURE 1:**
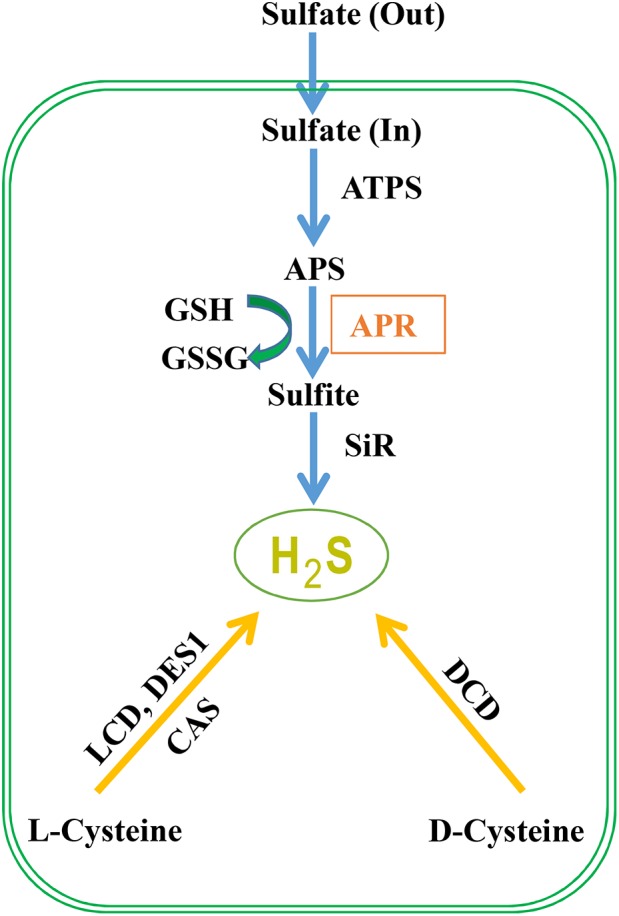
H_2_S metabolism in plants. Sulfate absorbed by plant roots through sulfate transporters is activated to APS (5′-adenylylsulfate) via ATP sulfurylase. APS can be reduced to sulfite by APR (APS reductase) which is further reduced to H_2_S by sulfite reductase. Subsequently, H_2_S is incorporated to cysteine (Cys), which is the first organic product of sulfur assimilation, and the starting point for methionine synthesis. Besides, cysteine is involved in the biosynthesis of coenzymes and vitamins including coenzyme A, biotin, thiamine, lipoic acid, and SAM (S-adenosyl-L-methionine) which is the donor of methylation. Besides the route of S-assimilation, H_2_S can also be produced from cysteine via the action of L-cysteine desulfhydrase (LCD), D-cysteine desulfhydrase (DCD), and DES1 which is member of O-acetylserine thiol lyase (OASTL) family proteins. Cyanoalanine synthase (CAS) generates cyanoalanine co-formed with H_2_S with cyanide and cysteine as the substrates.

Sulfate from the soil is transported by a proton/sulfate co-transport mediated by sulfate transporters in root epidermal cells, which subsequently is loaded into the xylem vessels and distribute it to the entire plant (Leustek et al., 2000). Then sulfate is stored into vacuoles or transported to chloroplasts to start the assimilatory pathway (Gotor et al., 2015). Before sulfate reduction, sulfate is activated to adenosine 5′-phosphosulfate (APS) catalyzed by ATP sulfurylase, and in *A. thaliana*, three chloroplast, and one cytosolic isoforms are discovered (Hatzfeld et al., 2000). Through two enzymatic steps located exclusively in chloroplast or plastid, APS is then reduced to produce H_2_S (**Figure 1**). The first step is the reduction of APS to sulfite by APS reductase (APR) using GSH as the reducing molecule. In the second step, sulfite is reduced to sulfide catalyzed by sulfite reductase in a six-electron reaction using reduced ferredoxin as reductant. APS can also be phosphorylated to 3′-phospho-APS (PAPS) by adenosine-5′-phosphosulfae kinase (APSK) to provide a sulfate donor for the modification of multiple natural products (Ravilious et al., 2012). Besides the sulfide production in sulfur assimilation, cysteine (Cys) could be degraded to generate H_2_S which is catalyzed by LCD, DCD, an O-acetylserine(thiol)lyase OASTL family protein DES1 or β-cyanoalanine synthase (an enzyme catalyzing the conversion of cysteine and cyanide to H_2_S and β-cyanoalanine) (Hatzfeld et al., 2000; Álvarez et al., 2010). Although the contribution of cysteine degradation to H_2_S has been highly valued, we propose that the influx of inorganic sulfur to cysteine is also crucial for H_2_S generation. The transport of sulfate into cells and the reduction of APS to sulfite by APR are the key regulatory steps of sulfate assimilation and APR is considered as the rate-limiting enzyme. Thus, the activity of the sulfur assimilation pathway controlled by APR may be required for animated response to changes in sulfur supply and to environmental stimuli that alter the need for reduced sulfur.

## Functional Domains in APR Proteins

Two domains of PAPS_reductase (adenosine 3′-phosphate 5′-phosphosulfate reductase) and Thioredoxin are found in APR proteins (Kopriva and Koprivova, 2004). The plant APR gene is believed to originate from a fusion between prokaryotic genes for APS or PAPS reductase and thioredoxin. The N-terminal domain (PAPS_reductase) constitutes the active center of APR, and C-terminal domain Thioredoxin is required for transferring electrons from GSH to the N-terminal domain (Bick et al., 1998; Kopriva et al., 2001). Despite that the sequence of the C-terminal domain of APR is more homologous to thioredoxin than to glutaredoxin, it functions as an efficient glutaredoxin (Leustek et al., 2000). Three independent steps are proposed for the reaction: the transfer of sulfate from APS to the active cysteine residue, the release of the sulfite by C-terminal domain, and the recovery of the active enzyme dimer by reaction with thiol. It has been found that plant APR contains an iron-sulfur cluster that is bound by the N-terminal domain (Kopriva et al., 2001), which is a decisive structure in the sulfate reduction pathway.

## Subcellular Localization of APR

Plastid or chloroplast is the only compartment that contains the enzymes for sulfur assimilation from sulfate to cysteine. Correspondingly, APR activity is localized in chloroplasts in spinach and in pea (Schmidt, 1976; Fankhauser and Brunold, 1978; Prior et al., 1999). Subsequently, the plastid localization of APR is confirmed in three *Flaveria* species by immunolocalization (Koprivova et al., 2001).

## Functional Characterization and Regulation of APR

As a rate-limiting enzyme, APR possesses a very high control over the flux through sulfate assimilation. It is reported that natural variation for sulfate content in *A. thaliana* is highly associated with *APR2* and a T-DNA insertion in *APR2* (*apr2-1*) induces more sulfate compared with wild type (Loudet et al., 2007). Besides, deletion of *APR* in *Physcomitrella patens* caused 50% reduction in the flux through sulfur reduction (Koprivova et al., 2002). Conversely, Arabidopsis overexpressing a *Pseudomonas APR* gene accumulates more sulfite, thiosulfate, cysteine, γ-glutamylcysteine and GSH, further confirming the important role of APR in sulfur assimilation (Tsakraklides et al., 2002). The metabolites in sulfur metabolism such as sulfite, H_2_S, cysteine and GSH are highly interconnected, thus interruption of the reaction from APS to sulfite carried out by APR will decrease the generation of H_2_S in plant. Therefore, we propose that the regulation of APR in sulfur assimilation might be critical for the dynamic H_2_S generation in response to environmental factors.

The uptake of sulfate and the reduction of APS by APR are controlled by the sulfur nutritional status of the plant. For instance, the three isoforms of APR in Arabidopsis are affected at expression and activity levels by the sulfur status, cysteine, GSH, O-acetylserine, nitrogen supply, sugars or phytohormones (Kopriva et al., 1999; Koprivova et al., 2000; Ohkama et al., 2002; Hesse et al., 2003; Kopriva and Koprivova, 2003). Exogenous addition of GSH to poplars not only increases GSH content, but also reduces APR activity and mRNA accumulation. GSH transport across the plasma membrane is a possible mechanism of the feedback regulation of sulfate assimilation by GSH. The postulation of a signaling GSH transporter, similar to the signaling hexose and sucrose transporters in sugar sensing (reviewed in Smeekens and Rook, 1997), would explain why APR is down-regulated only by exogenously added GSH. Also, GSH has been shown to be a negative signal for the regulation of APR expression in *A. thaliana* root cultures (Vauclare et al., 2002). H_2_S, an intermediate and also a signal in sulfur assimilation, induces a rapid increase in thiol compounds in the shoot of *Allium cepa* L. and *Brassica oleracea* L. and results in a down-regulation of APR activity and its mRNA levels (Durenkamp et al., 2007). In poplar trees, sulfur limitation induces an increase in mRNA levels of ATP sulfurylase, APR, and sulfite reductase, probably as an adaptation mechanism to increase the efficiency of the sulfate assimilation pathway (Kopriva et al., 2004). When the seedlings of curly kale (*B. oleracea* L.) are transferred to sulfate-deprived conditions, the expression of sulfate transporters and the activity and expression of APR are induced in roots and shoots respectively, suggesting that external sulfate status is the sensing factor for the modulation of sulfate uptake and its reduction (Koralewska et al., 2009).

Sulfur-containing compounds especially GSH play vital roles in plant response to stress conditions. Thus APR, a rate-limiting enzyme in cysteine synthesis, is closely related to GSH synthesis which is primarily dependent on the availability of the constituent amino acids. NaCl stress increases APR activity and its mRNA levels by 3-fold of all three isoforms in *A. thaliana*, but the response of APR to salt stress is independent of abscisic acid (ABA) and the induction of APR activity is not essential for the increase of GSH synthesis after salt stress (Koprivova et al., 2008). APR also plays a key role in plant response to selenate toxicity. A mutant Arabidopsis line (*apr2-1*) shows decreased selenate tolerance and photosynthetic efficiency, accompanied with an increase in total sulfur and sulfate and a 2-fold decrease in GSH concentration (Grant et al., 2011).

Phytohormones also affect sulfate uptake and assimilation. The key enzyme APR is regulated by ethylene, ABA, NO and other hormones (Koprivova and Kopriva, 2016). Ethylene synthesis and sulfur metabolism are closely connected through S-adenosyl-L-methionine (SAM) and methionine salvage cycle. Feeding of ethylene precursor 1-aminocyclopropane-1-carboxylic acid (ACC) can up-regulate APR activity by increasing mRNA levels of *APR1* and *APR3* in Arabidopsis (Koprivova et al., 2008). Zeatin is found to increase transcript accumulation of *APR1* and *SULTR2;2*, as a general effect on sulfate assimilation (Ohkama et al., 2002). Salicylate also induces the expression of all three *APR* isoforms and enzyme activity in Arabidopsis (Koprivova et al., 2008). Moreover, *APR2* expression is regulated by light/dark cycles and sucrose feeding (Kopriva et al., 1999).

Previous researches show a strict correlation between APR mRNA levels, protein accumulation and enzyme activity, suggesting that APR is primarily regulated at the transcriptional level (Kopriva et al., 1999; Kopriva and Koprivova, 2004). *APR* expression is activated by the MYB transcription factors MYB28 and MYB51 in Arabidopsis (Yatusevich et al., 2010). The bZIP transcription factor LONG HYPOCOTYL 5 (HY5) is also found to regulate the expression of *APR1* and *APR2* instead of *APR3* in response to dark adaption and sulfur status in Arabidopsis (Lee et al., 2011). Thus, the coordinated expression of *APR* by HY5 and MYB transcription factors links the regulation of sulfate assimilation to plant responses to environmental stimuli such as light, sulfur availability, and stress conditions.

Besides, modulation of APR activity via posttranslational redox regulation has been demonstrated in Arabidopsis subjected to oxidative stress, which provides a rapidly responding, self-regulating mechanism to control GSH synthesis (Bick et al., 2001). Salt stress is found to induce *APR* mRNA levels, whereas its protein accumulation is not observed, suggesting a likely translational regulation to control APR activity (Koprivova et al., 2008). The activity of APR is also regulated by the reduced form of sulfur through the transition from active dimer to inactive monomers (Kopriva and Koprivova, 2004). MicroRNA miR395 is found to target sulfate assimilation pathway by recognizing a low affinity sulfate transporter *SULTR2;1* and three *ATPS* genes *ATPS1*, *3* and *4*, whereas *APR* is not targeted by miR395 (Jones-Rhoades and Bartel, 2004; Allen et al., 2005). Whether there are other microRNAs regulating *APR* apart of miR395 or whether APR is subjected to protein modification such as phosphorylation is still unclear and needs further research.

## Future Perspectives

The regulation of APR in sulfur assimilation and H_2_S generation is complex which involving many signals and effectors. The sulfur-responsive *cis*-element SURECOREATSULTR11 is sufficient and necessary for sulfur deficiency responsive expression of the high-affinity sulfate transporter gene *SULTR1;1* in Arabidopsis roots, which could be reversed when supplied with cysteine and GSH (Maruyama-Nakashita et al., 2005). *APR3* (At4g21990) in Arabidopsis containing SURECOREATSULTR11 *cis*-acting element also shows response to sulfur deficiency conditions (Maruyama-Nakashita et al., 2005; Henríquez-Valencia et al., 2018). However, although APR is highly regulated in plants, transcription factors responsible for this regulation are still not fully understood. Bioinformatic approaches predict that gene expression response to sulfur deficiency is regulated by a limited number of transcription factors including MYBs, bZIPs, and NF-YAs, indicating that these transcription factors may play important roles in the sulfur status response (Henríquez-Valencia et al., 2018). Besides, other *cis*-regulatory elements are found in the promoter region of *SlAPR* genes, which may response to distinct plant hormones, such as cytokinins, and several environmental factors, as well as CO_2_, light, and abiotic and biotic stresses. Thus, considering the central role of APR in sulfur assimilation and possibly in H_2_S generation, how it is regulated by environmental signals and phytohormones still needs further research.

## Author Contributions

YF, JT, G-FY, Z-QH, Y-HL, X-YC, K-DH, and HZ conceived the paper. YF, G-FY, L-YH, K-DH, and HZ wrote the paper. All authors have read and approved the manuscript.

## Conflict of Interest Statement

The authors declare that the research was conducted in the absence of any commercial or financial relationships that could be construed as a potential conflict of interest.
